# Quieted City Sounds during the COVID-19 Pandemic in Montreal

**DOI:** 10.3390/ijerph18115877

**Published:** 2021-05-30

**Authors:** Daniel Steele, Catherine Guastavino

**Affiliations:** 1 School of Information Studies, McGill University, Montreal, QC H3A 1X1, Canada; catherine.guastavino@mcgill.ca; 2 Centre for Interdisciplinary Research in Music Media and Technology, Montreal, QC H3A 1X1, Canada

**Keywords:** environmental noise monitoring, urban sound environment, festival management, COVID-19, acoustic indicators, sound levels

## Abstract

This paper investigates the transformation of urban sound environments during the COVID-19 pandemic in Montreal, Canada. We report on comparisons of sound environments in three sites, before, during, and after the lockdown. The project is conducted in collaboration with the Montreal festival district (Quartier des Spectacles) as part of the Sounds in the City partnership. The analyses rely on continuous acoustic monitoring of three sites. The comparisons are presented in terms of (1) energetic acoustic indicators over different periods of time (L_den_, L_d_, L_e_, L_n_), (2) statistical acoustic indicators (L_10_, L_90_), and (3) hourly, daily, and weekly profiles of sound levels throughout the day. Preliminary analyses reveal sound level reductions on the order of 6–7 dB(A) during lockdown, with differences more or less marked across sites and times of the day. After lockdown, sound levels gradually increased following an incremental relaxation of confinement. Within four weeks, sound levels measurements nearly reached the pre-COVID-19 levels despite a reduced number of pedestrian activities. Long-term measurements suggest a ‘new normal’ that is not quite as loud without festival activities, but that is also not characterizable as quiet. The study supports reframing debates about noise control and noise management of festival areas to also consider the sounds of such areas when festival sounds are not present.

## 1. Introduction

### 1.1. Sound Monitoring and COVID-19

In early 2020, the COVID-19 pandemic spread worldwide. Local, regional, and national governments stepped in to attempt to control the spread of COVID-19 with varying degrees of action, ranging from inaction to different levels of restrictions, to near-complete lockdowns. These restrictions and lockdowns disrupted traffic patterns, commercial activities, and social and cultural events in ways that are unprecedented. Naturally, these changes in human activity patterns had an observable effect on urban sound environments, often cited in public discourse and the media, but also motivating a wealth of impromptu research questions and reframing existing ones.

A handful of researchers in cities around the world were prepared to study these changes to the sound environment thanks to a rise in urban noise monitoring systems. The Quartier des Spectacles (QDS) is the site of one of these permanent noise monitoring systems, which began to be installed in 2019 by the not-for-profit organization *Partenariat du Quartier des spectacles*. In the context of the QDS, a neighborhood where in pre-pandemic years, the bulk of Montreal’s large culture, festival, and public space activities were hosted, the changes to the sound environment were drastic. In the center of Montreal, the overlapping restrictions resulting from the COVID-19 pandemic manifested in a gradual shutdown starting in March 2020, continuing through 2021, in various manifestations (summarized below). This paper addresses the observed changes in Montreal’s sound environment in the QDS throughout a year of restrictions and it attempts to contextualize these changes in contrast with the pre-pandemic conditions of 2019. We also add to an emergent literature on what happened to pandemic sound environments after the lockdowns, but while many other restrictions were still in place.

We first detail the unique context of QDS and afterwards offer an overview of literature published so far on potential aspects that have influenced variations in sound levels in different cities around the world, in order to set the stage for the comparison of sound levels presented in the Montreal case.

### 1.2. QDS Context

The QDS is a mixed-use neighborhood in downtown Montreal of approximately one square kilometer that includes festival areas as well as a major university campus, libraries, dining, bars, and housing. The Place des Arts complex, which includes multiple concert halls and theaters, is the centerpiece of the district. The QDS has eight public spaces with cultural programming throughout the year, the highest capacity of which is the Place des Festivals. At any given time, the QDS can welcome as many as 30,000 indoor seated guests and more than 100,000 outdoor guests such as festivalgoers attending QDS’s year-long festival season, with the May–October period hosting festivals attracting the largest crowds. When not used for major festival stages, the Place des Festivals is used to hold smaller, year-round concerts and shows in addition to non-concert activities, such as the Montréal en Lumiere light festival or to serve as a large public fountain using floor-level water jets. While only broad information is available, estimates suggest that 12.000 residents live in the neighborhood (a number that is growing), with 45.000 jobs available and around 50.000 students using the many educational buildings (Parteneriat du Quartier des Spectacles 2019). For many years the Partenariat has adhered to strict rules requiring all outdoor events to end by 11 PM, and this ‘curfew’ is well known throughout the city and neighborhood. While the Parteneriat carefully manages the festivals through such rules and as shown by the installation of the sound level monitoring network described in the present study, their combination of noise management strategies appears to be paying off; emerging research documents how many residents of the QDS are not only happy with rather than annoyed by the presence of festivals in their neighborhood, they are thriving in the unusual sonic circumstances of the festival sound environments, considering themselves as “privileged spectators” [1].

## 2. Review

COVID-19 brought with it a reduction in a diverse array of human activities [2] leading to changes in the sound environment, observed around the world. In addition to extensive media reports [3] detailing the ‘quiet’ and ‘silence’ of the original COVID-19 lockdowns, scholarly literature is also beginning to emerge on changes to the broader environment. These ranged from changes in animal species behavior [4] to reduced global pollution [5]. Analogously “noise pollution” has decreased, thanks to associated reductions in transportation, industry, and recreation that also led to decreases in the noise associated with these sources, described in Section 2.2 below.

### 2.1. Types of Indicators to Measure Changes to the Sound Environment

To better characterize urban acoustic environments, measures of sound pressure levels can undergo an array of operations, from frequency weighting to time averaging. Standard measures calculated from the sound pressure are referred to as ‘acoustic indicators’.

Generally, indicators can be grouped into two broad categories: energetic and statistical. Energetic indicators include A-weighted equivalent continuous pressure levels (LA_eq,T_), where T denotes the time period over which the fluctuating sound levels are averaged (e.g., LA_eq,24hours_) or periods within a day (L_d_, L_e_, L_n_, respectively for day evening and night). Energetic indicators can be corrected for certain time periods including the day evening night indicator (L_den_) with a penalty for night (+10 dBA) and evening (+5 dBA). Statistical indicators, on the other hand, measure the sound level exceeded for a certain percentage of the measurement period (L_10_ is the upper 10th percentile of the signal, used to estimate emergent sounds; L_90_ is the upper 90th percentile, used to estimate background noise). Many of these indicators are required by noise policies including the European Noise Directive [6].

In this paper, we make use of both the dBA and dBC frequency weightings to support a frequency-based analysis. The A-weighting accounts for changes in human hearing sensitivity as a function of frequency by applying negative gains below 1 kH and above 8 kHz to approximate the frequency response of the human ear. It is commonly used for environmental noise measurements but potentially underestimates the impact of low frequency content. The C-weighting, based on the sensitivity of the human ear at high levels, relies on a flat filter between 31.5 Hz and 8 Hz. It is used for traffic, mechanical and entertainment noise. All indicators used in this study are otherwise defined in Table 1 below.

### 2.2. Sounds of COVID-19 in Other Cities

Several studies have been conducted around the world to document the effect of COVID-19, particularly lockdowns, on the sound environment. We summarize the findings of a review of 19 English and French papers published up until 1 March 2021; however, select relevant articles were included on an ad-hoc basis after this specified date. Papers were identified for characterizing the sonic dimension in relation to COVID-19. To our knowledge, no longer-term studies have yet been published detailing the extended effects of COVID-19 on urban sound environments in the months post-lockdown as restrictions were easing and as second waves of infection brought new, modified restrictions and other public health measures.

We begin by detailing overall (mean) sound level reductions, followed by a description of observed reductions in traffic and airport noise, for which some of these monitoring systems were purpose-built. These sections are followed by descriptions of some of the factors that influence the extent of the changes to the sound levels, including the urban morphology and program (space function), whether it is nighttime or weekend, the stage of the lockdown. Finally, a short section on perceptual and experiential changes to the COVID-19 influenced soundscape concludes.

#### 2.2.1. Mean Sound Level Reduction

Measures of mean noise reductions for cities where such figures were reported were usually at least an order of magnitude (using usually L_den_ or 24-h LA_eq_ measurements): 2–3 dBA in Stockholm, Sweden [7]; 4–6 dBA in Lyon, France [8] and London, UK [9]; 6–8 dBA in Milan, Italy [10], and Paris, France [8]. The reductions also produced effects that could be measured in terms of populations, such as an Italian study showing that 60% fewer people were living at L_den_ levels greater than 65 dB than had been living at these levels for previous years [10].

#### 2.2.2. Reduction in Traffic Noise

Naturally, traffic circulation was significantly reduced in city centers. In Sydney, Australia, traffic was down 52% on arterial roads and 81% on central shopping streets. Measurement stations around France detected a 4–6 dB reduction in traffic noise, which is about a 60–75% energy reduction, and was consistent with government reports on the scale of reduction in traffic volumes also having been reduced in this 60–75% range [11]. In terms of pedestrians, Melbourne, Australia released data showing that pedestrian use was only 14.6% of 2019 pre-pandemic levels in its Central Business District [12]. One important facet to note about the reductions in overall traffic volumes however is that average vehicle speeds actually increased in many areas due to the roads being more open. Cities such as Rome, Italy saw small increases in measured sound levels next to arterial roads due to this effect [13]. In cities with more wide-ranging curfews, such as in Lima, Peru, a neighborhood near the main international airport reached record low overnight values of around 35 dBA due to flight restrictions on top of the near complete elimination of traffic [14]. Thus, the changes in COVID-19 sound levels emerging from traffic borne-noise seem to be dependent on the local context, such as the strength of the lockdown and the comparative use of roads. Sound level reductions in cities that had overall fewer COVID-19-related restrictions had, in turn, smaller reductions in overall sound levels, such as Stockholm and Kobe, Japan [7,15].

#### 2.2.3. Reduction in Airport Noise

In terms of airports, while Paris at-large saw a mean reduction of 7.6 dBA (L_den_), the changes were much more significant at measurement stations around the Charles de Gaulle and Orly airports (up to 20 dB), with a consequent drop in noise complaints stemming from air traffic [16]. Athens, Greece showed similar differences across its network of 14 sensors, such that mean reductions varied between 3–6 dBA-L_den_, with a mean of 6 dBA-L_den_ near airport-adjacent highways, and up to 10 dBA-L_den_ near the airport. The Greek study was also able to demonstrate statistically different levels between their two control year conditions of 2018 and 2019, and that air traffic had a stronger lowering effect than road traffic [17].

#### 2.2.4. Spatial Considerations

Within each city itself, changed in measured sound levels were not consistent in place and time—a few variables have emerged as key indicators, with literature quickly emerging on the topic suggesting that different types of urban morphologies experienced changes to different degrees. A study of 11 London sites reported a mean drop in level across all sites of 5.4 dB (L_eq_), which ranged from a low 1.2 dB to a many-fold decrease of 10.7 dB. A detailed clustering analysis revealed three categories by which sound environments changed: (1) arterial roads saw a reduction of 3–4 dB, (2) parks, such as large parks and pocket parks, had about a 4–5 dB decrease, and (3) in areas with water features that dominate, there were only small 1–2 dB differences [9].

In the same London study, major tourist areas saw the most reduction of any sites: 8–10 dB. L_90_ and L_10_ measures showed similar changes to the LA_eq_ [9], suggesting a drop in both eventful sounds and background sounds. A study from Grenada, Spain showed a much more drastic change in sound levels at four tourist sites. The ranges of sound level reductions were 13–30 dBA; however, these were only made using single measurements for each condition. The presence of a water feature (i.e., a nearby river) maintained one of the four sites at a higher background level, based on L_90_ measurements. They found also a dramatic decline in low frequency sound and a 60–80% reduction in modeled loudness at these four tourist sites [18].

#### 2.2.5. Temporal Considerations

Time of day also emerged as a key moderator of the effect of the lockdown. Measurement stations near bars in France showed drops of over 10 dBA at night compared to pre-pandemic conditions, a far greater drop in levels than the 4–6 dB measured during the daytime and evening at most stations in their network [11]. In terms of traffic, rather than human sounds, a study of 24 measurement sites in Milan, Italy showed that during a normal pre-pandemic year (such as 2019), there would be two clear clusters of data for each day and night, but the lockdown of 2020 smoothed the difference between night and day, suggesting also less peak-period travel, previously associated with job-related commutes [19].

The time of the week was also important. A related study from the same Milan sensor network showed a 90% drop in traffic volume on Sundays compared to mean values between 50 and 70% on other days [10]. A separate study of five locations in Madrid showed that the reduction was unequal across times of day and for different urban morphologies, with markedly lower levels on weekends. The study highlighted a park (Casa de Campo: adjacent to a zoo and amusement park) and showed that while levels were normally higher on weekend afternoons and evenings, the long plateau from those periods disappeared during their lockdown [20]. A study using six measurement points in Kobe (where there was a less strict State of Emergency in place, rather than a lockdown) showed a difference between weekdays and weekends, with weekday mornings being louder [15].

#### 2.2.6. Type of Restrictions

Within the range of restrictions themselves, the stage of each city’s response (e.g., lockdown, quarantine, easing of restriction) played a role in sound level. Sensors in Lima indicated that the original flight restrictions and social distancing measures dropped the sound levels to 8 dBA below normal, but once a nighttime curfew also restricted evening activities and automobile traffic, it dropped to 12 dBA below normal [14]. Typically, sound levels returned ‘close’ to pre-pandemic levels after a few weeks since the beginning of the lockdown. France saw its levels begin to rise toward pre-pandemic levels around the sixth week of their lockdown [8]). Stockholm’s levels peaked at 4 dBA below pre-pandemic levels at the height of their mild restrictions, and returned to within 0.5–2.0 dB of normal pre-pandemic by June, as there was a rise in holiday gatherings associated with their mid-summer festivities [7]; similarly Italy’s sound levels rose but however did not return to pre-pandemic levels [21].

#### 2.2.7. Festival Sounds and the COVID-19 Sound Environment as-Experienced

One known study looked at the effect of COVID-19 on festival sounds. In Mumbai, India, religious festival participants were encouraged to celebrate at home, meaning that festival areas we approximately 28 dBA quieter than the same locations during religious festivals in 2018 [22]. These festival sounds were conceived as noise pollution; however, we take a different positionality in light of the aforementioned forthcoming study showing that residents of the district do not conceive of the Montreal festival sounds as negative. We do however, question emergence, or what sound sources are audible after the festival sounds have been taken away.

Previous studies pointed out this emergence of previously less audible sounds in the absence of typical city noises, such as birds [11] and Rome’s famous Trevi fountain being more distinguishable and more audible further away than in previous years [23]. Conversely, this new audibility increased noise complaints in New York City from emergency sirens [12]. While annoyance complaints about single sources may have risen, a study from Dallas, USA found an overall 14% drop in noise complaints, with those complaints from the city center explaining the majority of that drop [24].

### 2.3. Cities after Lockdown

Considering that the pandemic is not yet over at the time of writing, scholarship is still emerging on the longer-term effects of the lockdown on cities and to what extent cities are returning to the pre-pandemic ‘normal’. Interestingly, in Lyon, despite no change in the lockdown measures, the researchers actually saw a turnaround in the overall sound levels starting at about the sixth week of these measures still being in effect [8]. The aforementioned study of Monza, Italy [21] demonstrated how in the post-lockdown phase, levels rose from their lockdown minimum, but did not quite return to 2019 levels. In Rome and Milan, these levels were calculated as returning to 94% of the pre-lockdown sound levels [10]. In Stockholm, these levels went 0.5–2.0 dBA below normal as national restrictions were eased and holiday gatherings were taking place [7]. The city of Sakagami, Japan experienced a temporary surge over normal levels after their state of emergency was relaxed, especially for morning, evening, and overnight, but these quickly stabilized to ‘normal’ over a few days or weeks [15]. Finally, data collected all over Spain concluded that despite a dip of 15 dB in overall sound levels during the tightest phase of lockdown, the return to ‘normal’ sound levels was “lightly” higher than pre-COVID conditions [25]. On the perceptual side, a neighborhood in Basque Country, Spain experienced a rise in descriptions of *eventful* and *loudness* as their lockdown measures were eased [26]. Eventful and loudness as perceptual measures are mentioned here, due to their potential relationship with acoustic indices such as L_10_ and LA_eq_.

### 2.4. Review Summary and Research Objectives

In summary, a few major variables affecting sound levels during and after the lockdown emerge from the literature include temporal considerations (nights versus daytime, weekdays versus weekends) but also specific turning points time with each lockdown as well as location and spatial considerations. The literature suggests that heavily touristed areas have been highly variable in the observed changes seen around the world. In most cities that reported such information, levels registered to values that were higher than during the lockdown, but usually lower than pre-lockdown and 2019 levels. These findings lead us to pose the following research questions in our QDS context:

*RQ1*: What were the immediate effects of the COVID-19 lockdown in terms of observed sound levels? What was the influence of location or time on these observed changes? Were similar effects observed on different sites within the QDS?

Given the unique context of QDS with its dynamic festivals, we propose two additional questions related to the summer and winter period, extending previous research beyond the immediate effect of the lockdown.

*RQ2*: How was the summer of 2020 different in terms of sound levels when compared to a 2019, a pre-pandemic festival year?

*RQ3*: How is the sound environment of winter 2021 different from the winter 2020 just preceding lockdown?

## 3. Methods

### 3.1. Equipment and Data Collection Context

In the context of setting up a long-term monitoring system for Montreal’s extensive festival season, the overseeing organization, the PQDS began installing permanent sound level meters in July of 2019. Beginning with only one of these devices, the plan was to install 12 such meters around the one square—kilometer district. New nodes were being installed throughout the data collection period. The measurement devices themselves are permanently installed Type 1 microphones from Larson and Davis (SoundAdvisor Model 831C, Depew, New York, USA) installed two to three meters off the ground.

**Figure 1:**
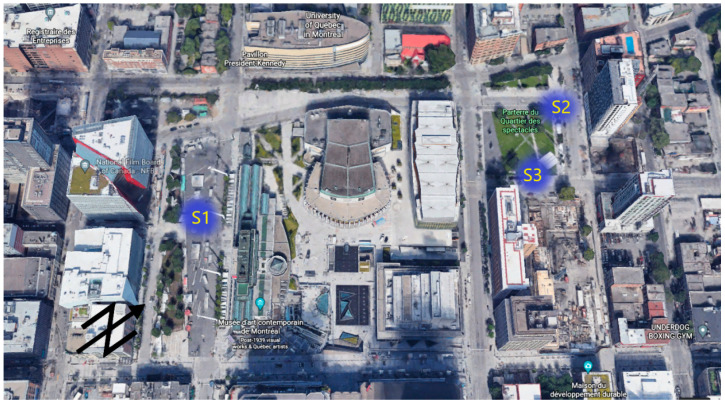
The center of the Quartier des Spectacles district with approximate measurement locations. Show are the main stage area (S1), and two separate areas within the Parterre, or secondary stage (S2, and S3). Additionally pictured is the local built context. Credit: Google Earth ©2021, image modified with labels by the authors.

Consequently, data from up to three measurement sites within QDS are used for the present study. The three sites represent a diversity of urban morphologies, traffic conditions, and public space use. The labels, locations, and measurement dates as well as a brief morphological description of each measurement station are presented in Table 2. A cartographical representation of these measurement stations is shown in Figure 1.

Data were retrieved from an online subscription portal, covering the timeline described in Section 3.2. All measurements provided are listed in Table 1 below. Pre-COVID-19 data are presented on a limited basis, as available. Additionally, where possible, we provide measurements according to the indicators proposed by [27], such as the percentage of days exceeding L_den_ = 65 and L_n_ = 55.

Sound level measures from the monitoring network are occasionally accompanied by observations from site visits to document the conditions.

### 3.2. Timeline

**Figure 2:**
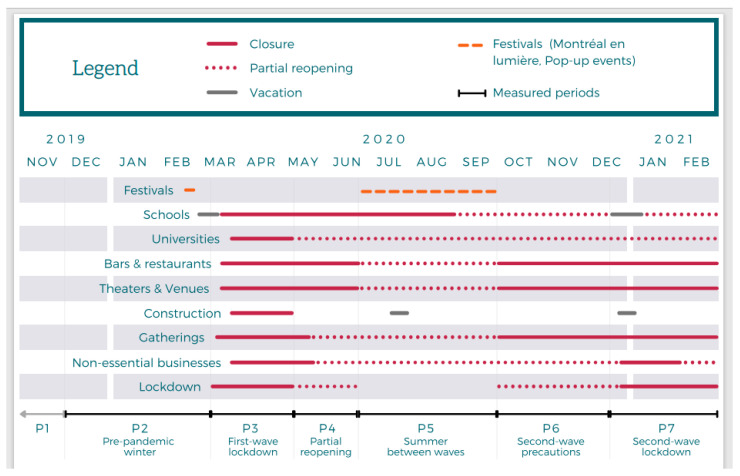
Timeline of key events and closures during the study’s time period.

The changes to Montreal’s QDS sound environment are measured by comparing over seven distinct periods, described below. References to specific key dates can be found in the accompanying text. For a visual depiction of the COVID-19-related timeline, see Figure 2. The periods are:

*P1: Pre-pandemic Festival Season* through November 2019

This period focuses on the summer and fall seasons of 2019. This measurement period establishes a baseline that includes normal operating conditions for the neighborhood including festivals.

*P2: Pre-pandemic Winter* 1 December 2019–9 March 2020

The winter period before the COVID-19 lockdown. This period is given a separate timeframe from the 2019 baseline because the harsh winter conditions of Montreal drastically limit outdoor activities in the neighborhood, including festival and construction activities. Nevertheless, a winter festival called Montréal en Lumière was held in the last two weeks of February. The festival involved a few evening-time concerts. Immediately following Montréal en Lumière, a provincial “study break” meant that all public schools, including those in the neighborhood, from primary to university, were on a break. This period may have contributed to an earlier decline in sound levels, which will be discussed in the results.

Key dates:

- 20 February–1 March 2020—Montréal en lumière festival
- 1–6 March 2020—Provincial study break

*P3: First-wave Lockdown* 10 March 2020–10 May 2020

The lockdown, as in many cities, was a gradual wave of tighter and tighter restrictions, beginning in the second week of March. Media reports indicated that only 8% of the typical number of visitors to the neighborhood were present [28].

Key dates:

- 12 March 2020—Government orders cancelation of all indoor gatherings over 250 guests
- 13 March 2020—Closure of schools and universities
- 14 March 2020—Closure of cinemas, theaters, libraries, museums
- 15 March 2020—Closure of bars and venues
- 21 March 2020—All gatherings banned, indoor and outdoor
- 22 March 2020—Closure of restaurants, salons, and shopping malls
- 25 March 2020—Government orders stop to non-essential construction activities

*P4: First-wave Partial Reopening* 11 May 2020–21 June 2020

The lockdown lasted fully for two months. One of the first non-essential activities to resume was construction and the reopening of some schools, which began on 11May2020.

Key dates:

- 11 May 2020—Construction permitting resumes and daycare schools set to reopen

*P5: Summer Between Waves* 22 June 2020–30 September 2020

This period saw a general easing of restrictions and greater use of public and some private spaces. However, all large festivals remained canceled and only small, unannounced pop-up events occurred.

Key dates:

- 22 June 2020—Montreal reopens indoor dining
- 24 June 2020—some theaters and venues reopen at reduced capacity
- 25 June 2020—bars, waterparks, casinos reopen
- 3 July 2020—Montreal nightclubs reopen under very strict conditions

*P6: Second-wave Precautions* 1 October 2020–16 December 2020

Montreal prepares for a second wave and introduces new restrictions. The province of Quebec declares Montreal a red-alert zone. Bars, restaurants, venues, cinemas close again

*P7: Second-wave Lockdown* 17 December 2020–1 March 2021

Schools send students home with a warning of stricter measures to come.

Key dates:

- 19 December 2020–3 January 2021—general school break
- 25 December 2020—full provincial lockdown. Closure of all non-essential businesses
- 9 January 2021—Montreal instates a curfew beginning at 8:30 p.m. each evening (later changed to 9:30 p.m., and again to 8:00 p.m.)

### 3.3. Analysis

The time periods identified above will be used as analysis windows to respond to the research questions.

1. To document the effects of the lockdown, we directly observe Period 3, especially with respect to time of day and time of week. Furthermore, we compare Periods 2, 3 and 4 to establish the decline and rise of the sound levels.
2. To establish the differences seen between summers and festival seasons, we continue by comparing Periods 1 to Periods 5 and 6.
3. To document the winter conditions, we compare Period 2 with Period 7.

## 4. Results

### 4.1. Effect of Lockdown at Three Different Locations

**Figure 3:**
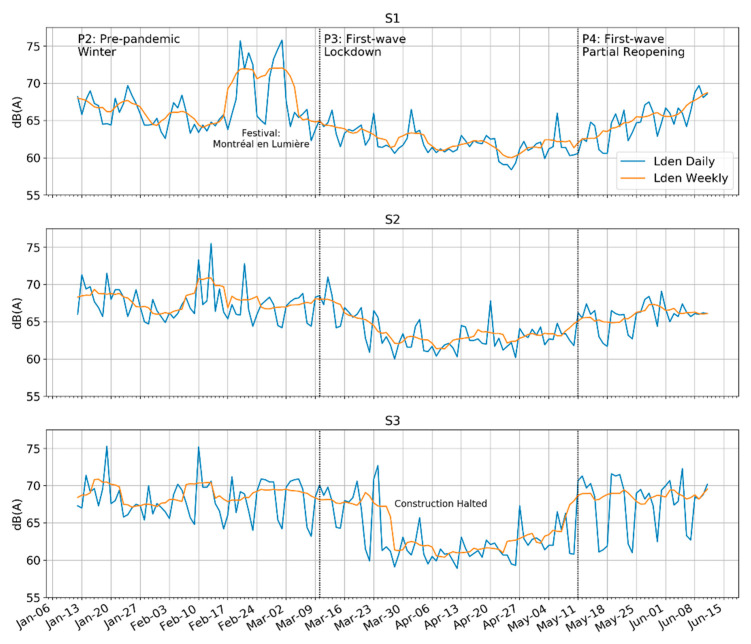
Daily L_den_ and superimposed weekly rolling average L_den_ across three sites in the Quartier des Spectacles (QDS). There is a clear ‘trough’ corresponding to the strictest measures associated with the Quebec/Montreal lockdown.

We begin the results by focusing on the daily L_den_ in Period 3, the lockdown. The data in Figure 3 show a clear trough spanning between two and three months, depending on the site of measurement, corresponding with the different public health measures. This is consistent with the stricter lockdown measures described in Period 3. While it is difficult to establish a baseline level in this area with diverse urban activities, the reduction in levels appears to be about 6–7 dBA at each of the three sites based on the rolling weekly average in comparison to Period 2 (*Pre-pandemic Winter*) outside of the festivals. The ‘bottom’ of the trough also amounted to between 60 and 62 dBA-L_den_ at all three sites. 

Comparing across the three sites, each had unique differences. At S1, there are two clear peaks just before the lockdown correspond with the Montreal en Lumiere festival. The long and gradual reduction in levels is consistent with the space with neither significant traffic nor significant human activity during P3. S2, next to a busier road with more erratic traffic and delivery patters, showed a longer and more gradual decline in levels, associated with the decline of many activities that were phased out during the early phase of the lockdown (Period 3). Finally, the sound levels at S3 dropped very suddenly compared to the others. This showed clearly that it was adjacent to a construction zone, which was not ordered to close until 25 March 2020, well into the lockdown. This is especially clear in the deep valleys corresponding with each weekend, especially in the periods when construction was allowed. There was a flattening of weekday and weekend levels throughout March.

#### 4.1.1. Day vs. Night

**Figure 4:**
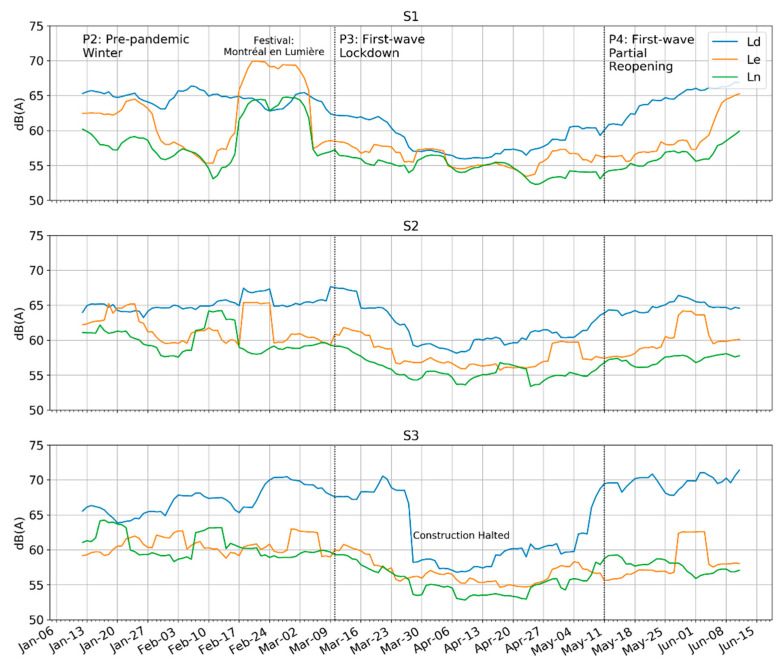
Daily L_d_, L_e_, and L_n_ (day, evening, and night) shows a sharper decline during lockdown (P3) in daytime levels compared to the evening and night.

While the L_den_ from the previous section adds the separately calculated equivalent levels for the three periods of day, evening, and night and applies a penalty for evening and nighttime levels, the individual levels for each period can be plotted separately to observe changes between them (see Figure 4). In the days just before 1 March 2020, the levels at S1 and S2, especially in the evening and night reflect the presence of the *Montreal en Lumière* festival that took place primarily in the evening and early nighttime hours (until 11 PM). In the presence of this and other evening and nighttime activities in the district it can be hard to determine a baseline sound level. Nevertheless, we can observe two different trends in this data: (1) that the L_d_ levels, which were usually the highest of the three, fell further than the other two; and (2) that the lowest points in the L_d_ did not correspond in time with the lowest points of the L_e_ and L_n_. The evening and nighttime levels continued to fall after the daytime levels had reached their minimum. This may be attributable to the staggered closures enacted by the government; however, it is not clear which measures corresponded to the falls and rises in the levels. The clear drop-off in daytime levels in the S3 data are most likely attributable to the 25 March closure of all construction activities, some of which were taking place adjacent to the measurement point. While compliance with the end of permitted construction was sudden, other measures that applied to the general public such as public gatherings probably required a slower trajectory of education, policing, and enforcement. See the timeline in the Method section (Figure 2).

#### 4.1.2. Weekday vs. Weekend

The previous analysis already showed evidence of a typical weekday versus weekend trend that was flattened by the Period 3 lockdown, where the weekends displayed lower levels pre-lockdown (P2), but the differences largely disappeared during the lockdown. Considering these changes, we present an in-depth analysis of the daily profile of multiple weekday and weekend days averaged within a representative window of time for each Period 2, 3, and 4.

**Figure 5:**
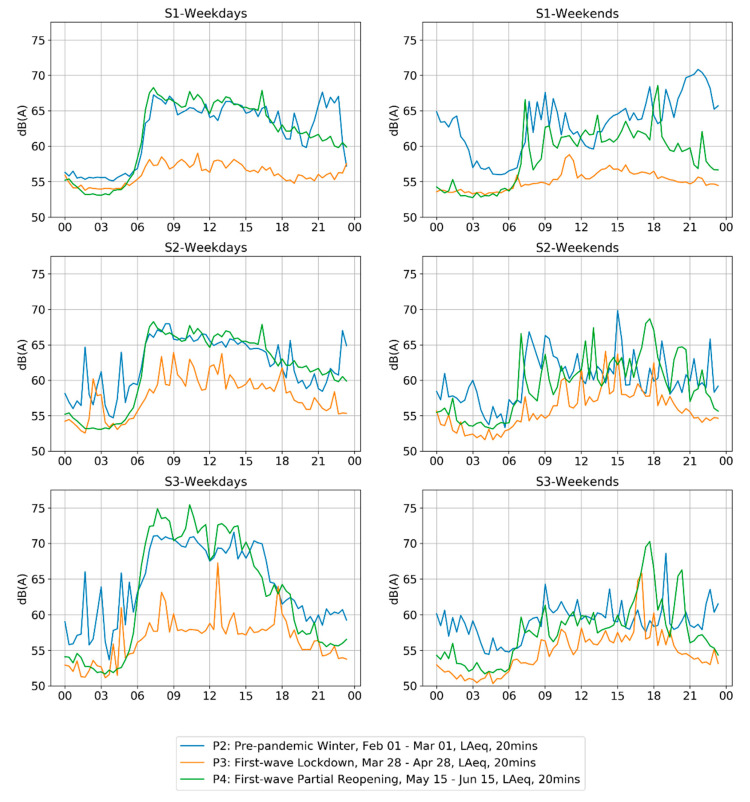
A comparison of levels throughout the day (L_eq,20min_) on weekdays and weekends, averaged across each weekday and weekend day over four representative weeks from each period (P2: Pre-pandemic winter P2; P3: First-wave Lockdown; P4: First-wave Partial Reopening. Analysis shows a clear difference in the daily profile between each condition. Distinctions between the weekday and the weekend that were previously clear are diminished.

Figure 5 shows the contour of LA_eq,20min_ averaged over weekdays (Monday–Thursday) and weekends (Saturday and Sunday) for four weeks in the middle of each period shown. Transition periods, including both Fridays, as well as the weeks where lockdown conditions were changing were left out.

On a pre-lockdown weekday, the levels begin to rise sharply around 6 a.m. During the lockdown, this morning rise still happened around the same time, but the change in levels was much smaller. After the lockdown (P4), the sharp morning rise and other aspects of the daily profile essentially returned to P2 levels, albeit with some minor variations. The midnight (12:00 a.m.–3:00 a.m.) peaks correspond to very loud events that took place in one of the 20-min periods, such as sirens from emergency vehicles.

On pre-lockdown weekend, a similar daily profile for the morning with a slow rise was observed; however, daily levels in the evening and at night reflect the neighborhood: high evening levels correspond to the Montreal en Lumière festival (particularly S1) and late-night levels correspond to the sounds of nightlife, most likely patrons exiting the closing bars approaching the 3 a.m. bar closing time. During the lockdown (P3), the daily profile more closely resembled the daily profile of a P3 weekday, meaning that the distinction between the weekday and weekend was less pronounced. During P4 on the weekend, the daily profiles resemble the weekend daytime from Period 2 albeit without any of the late evening and late-night festival and bar noise, as these activities were not allowed during Period 4.

#### 4.1.3. Frequency

**Figure 6:**
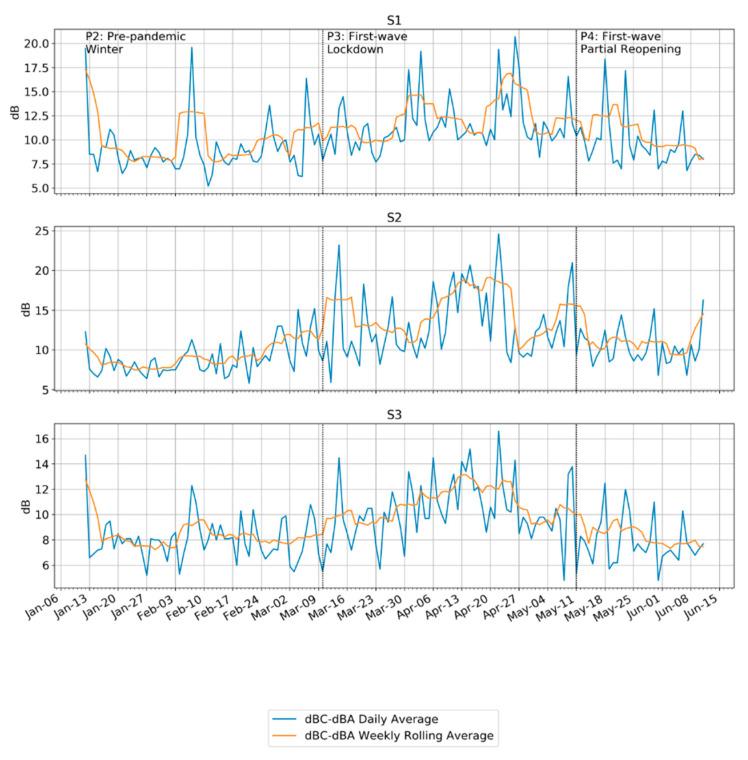
Comparison of background (L_90_) and emergent (L_10_) statistical indicators throughout the lockdown period (P3). Emergent sounds declined more than background sounds during P3; data from P2 and P4 are shown for context.

Up to this point, the measurements have used the dBA frequency weighting, which filters low frequency content, especially below 500 Hz. Generally, the difference between the same measurement taken with the A- and C-weighted filter (dBC–dBA) has been used as a shorthand for the amount of low frequency energy in a signal that could be attributable to traffic noise or mechanical equipment. Consistent with news reports stating that road traffic levels were around 10% of their pre-pandemic volumes, we would expect a large decrease in dBC–dBA. The resulting differences in 24-h equivalent and weekly rolling average sound level measurements are shown in Figure 6, using the same measurement period as in the previous figures (P2–P4). Contrary to expectations, the figure shows a rise in this difference, meaning that other sources of low frequency noise are likely present.

An analysis of sound recordings taken by these devices and soundwalks taken by the researchers confirmed the suspicion that the sound was attributable to the HVAC systems present on the very large buildings in the area, including a museum, symphony hall, opera house, shopping complex, hotels, and large residences. The loudness of these machines is something that will be revisited in the discussion.

#### 4.1.4. Background Sounds and Statistical Indicators

**Figure 7:**
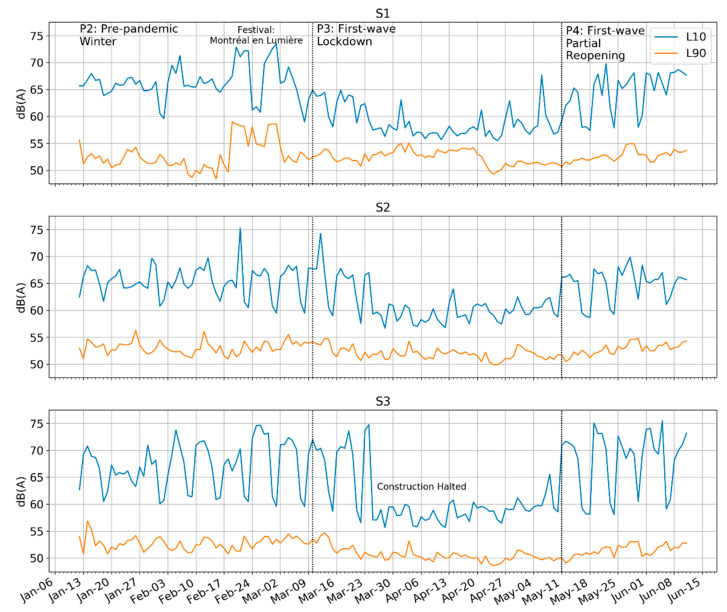
Comparison of background (L_90_) and emergent (L_10_) statistical indicators throughout the lockdown period (P3). Emergent sounds declined more than background sounds during P3; data from P2 and P4 are shown for context.

The statistical indicators (L_10_, L_90_) lend further support the idea of the presence of high background sound levels. Figure 7 plots the daily values for various statistical indicators throughout Period 3. Across all three sites, there is a noticeable flattening of L_eq_ and L_10_ sound levels while the L_90_ levels remain steady. This indicates that the sound environment used to be full of louder punctual sounds, including both construction and eventful human sounds, but that those largely disappeared during the lockdown.

**Figure 8:**
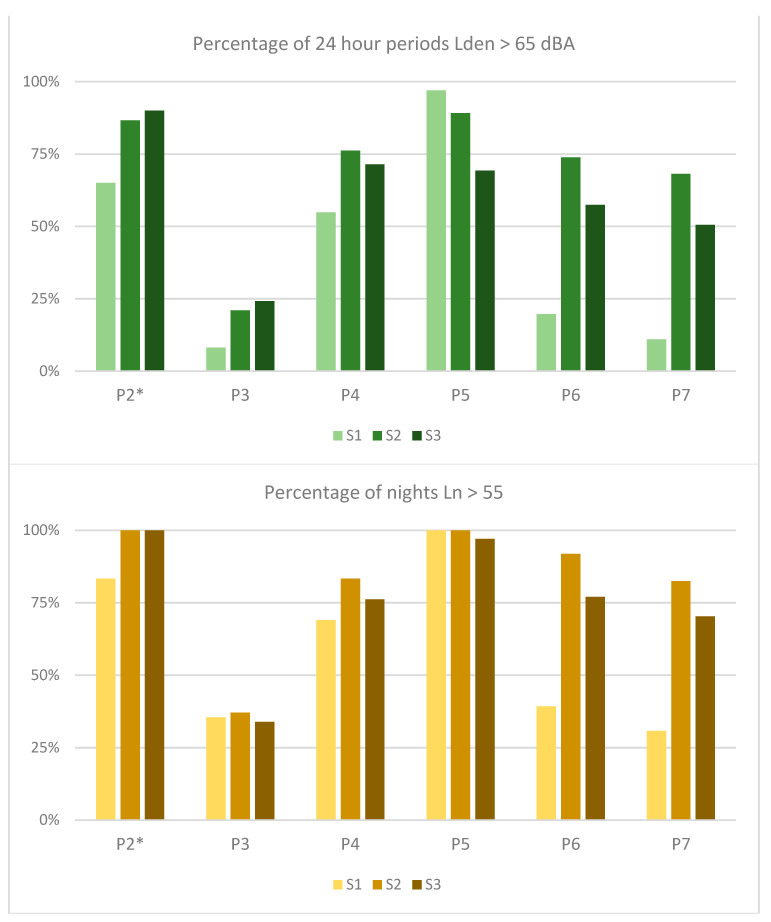
Percentage of days by site and by time period where L_den_ > 65 dBA and where L_n_ > 55 dBA. * Please note that P2 data does not cover the whole period due to the measurement network being offline.

Consistent with the changes experienced in other cities and to understand the extent to which the three sites experienced different changes in levels, the number of days (expressed as a percentage of days within a predefined period) where L_den_ exceeds 65 dBA and L_n_ exceeds 55 dBA is shown for each site in Figure 8. For the present results, we focus on the P2–P4 data. In Period 2, the weeks before the lockdown, the percentage of ‘noisy’ days and ‘noisy’ nights was high, and even approaching 100%.

The differences in L_den_ > 65, especially between sites, is further evidence of the unequal effects spread over a small urban area. Surprisingly, it was not the main festival stage area where the percentage of high L_den_ or L_n_ rates were the highest, and where those levels returned to the highest after the toughest restrictions were ended. Interestingly, the percentage of high L_n_ days during Period 3 was roughly the same across the three sites, narrowly ranging from 34–37%.

Given that consistently across the three sites the L_den_ reached between 60 and 62 dBA, and the percentage of L_n_ > 55 being around 35%, it is worth asking: what was the ‘background noise’ of the locked-down city center? In the absence of the 3 a.m. bar closing noise, why did the levels not fall further?

Construction noise, outside of projects with special permits, ends by law at 7 p.m. in the City of Montreal, yet in practice ends much earlier, around 3 p.m. The fact that construction was among the last things to be shut down on the lockdown timeline (25 May) and among the first things to be re-permitted (11 May), these levels created an ‘inner trough’ visible for Site S2 and S3 (see Figure 4 and Figure 7)

In conclusion, for RQ1, we see a precipitous decline in overall sound levels during lockdown (P3). The declines are unevenly spread across time (hour, period of the day, and day of week), site and frequency. The decline is more pronounced for emergent sounds (L_10_) than background sounds (L_90_). The resulting contours corresponded to a general flattening during P3 of sound levels spread across hours of the day, between weekdays and weekends, and a reduction in emergent sounds, such that there was less distinction between these periods than there was pre-lockdown. The ‘trough’ of the data were characterized by a high level of background noise, likely mechanical noise that dominated the altered sound environment.

### 4.2. Comparison between the Summers of 2019 and 2020

This section relies primarily on a comparison of Period 1 (summer 2019) to Period 5 (summer 2020).

**Figure 9:**
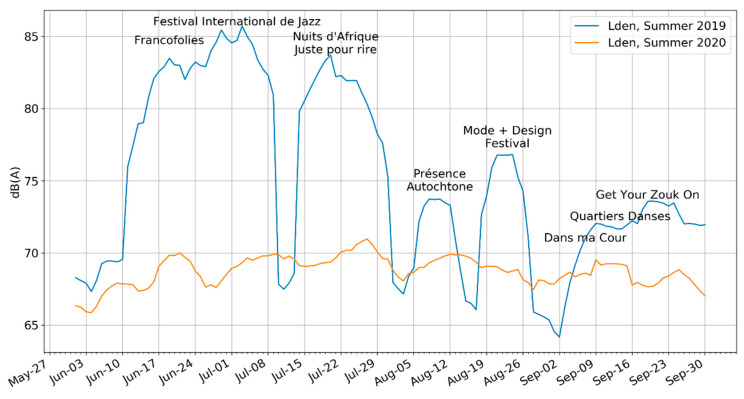
Sound levels comparing the summer of 2019 with 2020, using a weekly rolling average L_den_. The summer of 2019 is dominated by festivals, especially considering that the festivals often take place in the evening, when the L_den_ applies a 5 dBA penalty on sound levels. In the absence of these festivals, the sound levels cannot be characterized as low.

The typical summer is characterized by a festival season that dominates sound levels (see Figure 9). Compared to the pandemic season without festivals, the weekly average L_den_ could be at points as high as 15 dBA higher. Consistent with the microphones being placed at the center of the music areas, the elevated sound levels capture exactly what was expected. Please note that the difference in levels is emphasized by the use of the L_den_ because most of the festivals have events in the evening when there is a 5 dB penalty attached to sound levels from 7 p.m. to 11 p.m. Additionally, note that the levels are represented using a rolling weekly average to smooth the curve, which makes the levels during festivals appear more continuous than they are.

Focusing instead on the 2020 levels, we can see that in the absence of festivals and most other activities, the sound levels of the summer are still quite elevated but much more constant over time. In addition to the aforementioned HVAC systems that were continuously present, construction remained present throughout the period, except for a two week ‘construction holiday’ at the end of July–note that while there was a modest decline in weekly average levels in late July 2020, the S1 microphone is not positioned next to any ongoing construction sites. Additionally, large public fountains were frequently left on in the vicinity of the S1 microphone. The organization running the QDS also hosted several public space events, albeit at low capacities, including unannounced ‘pop-up concerts and small street performances to animate the neighborhood while discouraging crowds. These took place mostly in the evenings and weekends. Some restaurant terraces were also open, and on some Sundays an ongoing protest passed through the center of QDS.

### 4.3. Comparison between the Winters of 2019 and 2020

This section relies primarily on an analysis comparing P2 (Pre-pandemic Winter) to P7 (Second-wave Lockdown), namely the winter periods of both 2020 and 2021.

**Figure 10:**
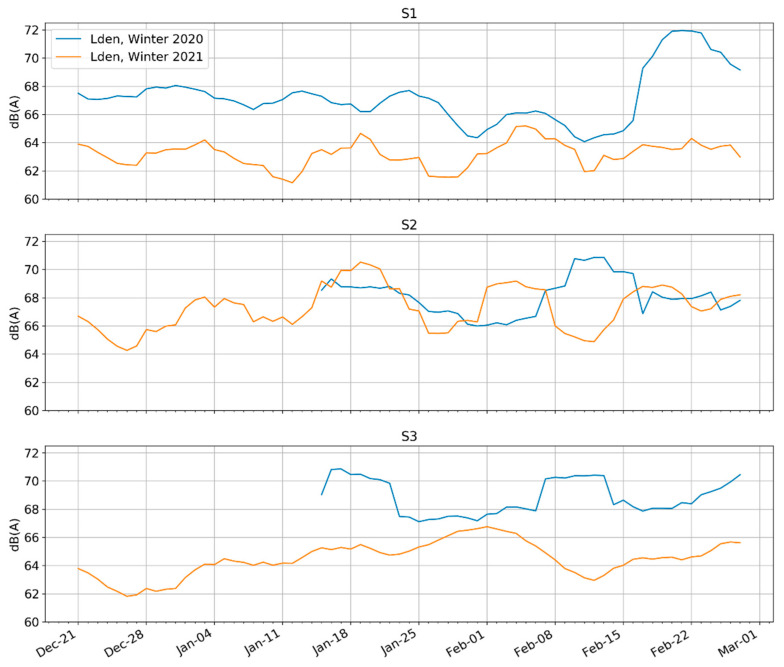
Rolling weekly L_den_ sound levels comparing Winter 2020 (P2) to Winter 2021 (P7). Please note that the data at S2 and S3 began getting collected in early January 2020.

Figure 10 shows the rolling weekly L_den_ from the end of Period 6. Please note that Quebec went into a modified lockdown on 25 December 2020, which did not have a clear effect on the measured sound levels. However, the school holiday extending from 19 December 2020 to 3 January 2021 may partly explain the low levels during this time. Furthermore, on 9 January 2021, Quebec instated a nightly curfew on its residents living in ‘red zones’, including all of Montreal, which began each evening between 8:00 p.m. and 9:30 p.m., changing frequently. L_den_ in the QDS did not appear to decrease in response to the curfew. Lastly note that the measurements were taken during Montreal’s harsh winter, when public life is not as consistent as it is in the summertime.

Depending on the site, the more recent 2021 sound levels were either consistent with the previous year’s (S2) or, they were as much as 5–7 dBA quieter (S1 and S3). The large drops in level at the S1 and S3 sites for 2021 may be attributable to the completion of major construction works on a pedestrian site, which ended on 26 November 2020. Returning to Figure 8, showing the percentage of days with L_den_ greater than 65 dBA and L_n_ greater than 55 dBA, we see that comparing P2 to P7 paints a consistent picture with the L_den_ data presented in Figure 10, namely that S1 and S3 saw higher drops. However, also referring to Figure 8, the L_den_ < 65 percentages of Period 6 were also low compared to Period 5; thus, it is possible that the October 1st closure of dining and other activities had a stronger impact on L_den_ levels than either the closure of construction or the curfew imposed during Period 7.

## 5. Discussion

We begin the discussion by returning to the three research questions in light of the other studies from around the world:

*RQ1*: In terms of overall sound level reductions, Montreal appears to be in the ‘middle of the pack’ compared to other cities. However, the unique festival schedule (including one festival one week before lockdown began) and harsh winter conditions are contexts that should be kept in mind. The Quartier des Spectacles had an approximately 6–7 dBA reduction in sound levels outside of the festival events, on par with Milan and Paris; higher than Stockholm and Lyon, and lower than London’s touristic areas [9]. In the comparable case of London’s touristic areas, the researchers documented a drop in both L_10_ and L_90_ levels, whereas in Montreal, the reduction was concentrated in the eventful L_10_ sounds, while background sounds (L_90_) remained elevated due to the HVAC systems.

As with other cities, in addition to having variation across multiple sites, there were differences in the effects of lockdown on days, evening, and nighttime sound levels and across weekdays and weekends (see Section 2.2.5). However, in contrast to seeing more pronounced differences between day and night [11] or weekdays and weekends [10], Montreal’s sites showed a flattening during lockdown between weekday and weekend distinctions and between day, evening, and night levels (as did [19]).

Here it is also worth noting that the minimum evening and nighttime levels only reached a low of about 55 dBA. In Lima, by contrast, the lowest nighttime levels reached 35 dBA [14]. The World Health Organization, for example, advocates an L_n_ (L_night, outside)_ of 40 dB as the target of the night noise guideline to protect the public, including the most vulnerable groups such as children, the chronically ill and the elderly. A L_night,outside_ value of 55 dB is recommended as an interim target for the countries where the night noise guideline cannot be achieved in the short term for various reasons [29]. The likely explanation of the high outdoor overnight sound levels is the presence of other sources that were not impacted by the lockdown, such as HVAC systems or other types of mechanical noise associated with the large buildings in the neighborhood. These sound sources should also be considered in the noise management of the QDS.

*RQ2*: In the absence of festivals, the sound levels could be characterized as being ‘as high as’ 15 dBA lower than pre-pandemic. The only study showing comparable changes came from the touristic areas of Granada [18], which had between a 13 and 30 dBA drop. The differences, however, were that in Montreal, the microphones were specifically monitoring the sound levels of loud festivals–in the absence of these festival activities, it was still a loud central urban area with human activities, construction, and other mechanical equipment. In Granada, the spaces were relatively desolate of alternate activities in the absence of tourists. Those measurements were also taken and compared during the strictest part of their lockdown, while those in Montreal were taken in the post-lockdown, but still restricted, summertime, where use of public spaces increased.

*RQ3*: The ‘new normal’. Over the weeks following the lockdown (P4), Montreal’s sound levels essentially returned to what appeared to be pre-pandemic levels. However, in the absence of this data network’s equivalent data from the 2019 winter period, it is impossible to know if those sound levels should have been higher than they were. To make the comparison to the sound levels of the neighborhood in the temporary new normal of early 2021, we compared the winter of 2020 with 2021. While the levels in Montreal approached 5–7 dBA during a period when there was an evening curfew, these reductions were far smaller than what was seen during Lima’s nighttime curfew, which was as high as 12 dBA [14].

## 6. Conclusions

To conclude, we saw a drastic reduction in sound levels in the downtown festival district, Quartier des Spectacles. A decrease of 6–7 dBA corresponds to about 25% of the noise emissions observed before the pandemic hit. Nevertheless, the residents and visitors to the district were not able to enjoy the levels of quiet that others in the world enjoyed because of mechanical noise permeating the area, and the resumption of construction activities after 7 weeks of lockdown. The lowest LA_eq_, 24 h levels rarely went below 55 dBA (neither below 60 dBA-L_den_, and 50 dB LA_eq, 20min_), even during the lockdown, far above WHO target levels.

The literature identified in the review was focused primarily on both sound levels and the various cities’ lockdowns specifically. More research is needed that looks beyond sound levels to document the changes as they were experienced by city users, primarily through qualitative and listening-oriented studies. Was the QDS lockdown experience consistent with the global media reports that described the time period as ‘quiet’ or a ‘return to nature’?

After the main lockdown (Period 3), the sound environment returned to a new “normal” that was almost as loud, but without any of the usual excitement of festivals. The post-lockdown Period (Period 4 and onward) saw the return of activities such as construction and sound sources such as automobile traffic. This is important to consider in the context of policy, suggesting that rather than focusing largely on the management of festivals and musical events, the municipality could, if it were focused on managing noise outcomes, consider more reflection on the mechanical sources of noise that overwhelm the neighborhood.

In terms of long-term management for the QDS, it is necessary to acknowledge that the festivals are loud in general. However, given our experience in the QDS without these festivals, should we still consider the festival to be the core focus of noise reduction and noise management?

A recent study [1] documents how residents of the QDS in a pre-lockdown summer thrived and enjoyed the use of their homes during the festivals; their main sources of noise concerns centered on construction and late-night bar use. Future studies building on this work will complement the sound level analysis with a qualitative approach to document how people used and experienced various Montreal public spaces throughout lockdown as well the summer afterwards, and how that changed their perception of sound, evaluation of various sound sources as well as their overall relation with both the outdoor urban sound environment and public space. Such studies are needed to investigate the perceptual and behavioral dimensions underlying the richness and complexity of city user experiences beyond decibel values.

## Figures and Tables

**Table 1:** We use the following indicators to characterize the sound environment.

Measure	Description
L_10_	Sound level exceeded 10% of the time—indicator of emergence
L_90_	Sound level exceeded 90% of the time, measures level of “background noise”
L_day_ (L_d_)	A-weighted equivalent continuous sound level over the period 7 a.m.–7 p.m.
L_evening_ (L_e_)	A-weighted equivalent continuous sound level over the period 7 p.m.–11 p.m.
L_night_ (L_n_)	A-weighted equivalent continuous sound level over the period 11 p.m.–7 a.m.
L_den_	A-weighted equivalent continuous cumulative exposure calculated from L_d_, L_e_, and L_n_. Corrections are applied by arithmetic addition to the levels measured in the evening (+5 dBA) and at night (+10 dBA). The levels of the three periods are then summed logarithmically
LA_eq,T_	A-weighted equivalent continuous sound level over time period T
LC_eq,T_	C-weighted equivalent continuous sound level over time period T
% days exceeding L_den_ = 65	Standard calculation for comparing cities
% days exceeding L_n_ = 55	Standard calculation for comparing cities

**Table 2:** Current measurement locations, earliest date.

Code	Location	Date of First Measurement	Site and Morphological Considerations
S1	Place des Festivals Streets: Balmoral and Mayor	24 May 2019	Site is the largest concert stage (capacity 25 k festivalgoers). In the middle of a large public square with fountains. During events, it is just off from the center of the audience area. A road passes through, which is often closed long-term for these events.
S2	Parterre North Streets: Clark and Maisonneuve	7 January 2020	A medium-sized green space bordered by busy roads. During the daytime, the intersection of a few busy roads, many buses, the confluence of bicycle paths. During festivals, the space is the host of large concerts. Microphone just off from the center of the audience area. Police headquarters and a nearby fire station mean the frequent passage of sirens.
S3	Parterre South Streets: Montigny and Clark	7 January 2020	On a lightly trafficked side of the parterre and more distant from any significant roads. Typically, just behind the stage for large concerts. This space directly adjacent to the site of a large development under construction.

## Data Availability

Restrictions apply to the availability of these data. Data were obtained from SETI Media and are available from the authors with the permission of the Partenariat du Quartier des Spectacles.
